# Outpatient reimbursement policies for cancer treatment in China: a comparative and longitudinal analysis

**DOI:** 10.1186/s12913-026-14418-0

**Published:** 2026-03-23

**Authors:** Haoqi Wei, Jinwei Zhang, Xingchen Liu, Xingyu Liu, Xiaoyong Liu, Shuchen Hu, Kaining Yan, Caijun Yang

**Affiliations:** 1 Department of Pharmacy Administration, School of Pharmacy, Xi’an Jiaotong University, No 76, Yanta West Road, Xi’an, Shaanxi Province China; 2 Center for Drug Safety and Policy Research, Xi’an Jiaotong University, Xi’an, Shaanxi Province China

**Keywords:** Outpatient reimbursement, Medical insurance, Cancer, Policy recommendation

## Abstract

**Background:**

Cancer poses a huge health and economic burden globally, with China being no exception. While China’s basic medical insurance (BMI) program has expanded outpatient coverage for chronic diseases, including cancer, there is a lack of national-level guiding policies. This study aims to investigate disparities and inequities in outpatient cancer reimbursement across China by analyzing reimbursement policies in different cities and under different medical insurance schemes.

**Methods:**

We conducted a cross-sectional analysis of outpatient reimbursement policies for cancers in China in 2022, supplemented by a longitudinal policy analysis. Data were collected from 85 prefecture-level cities under two insurance schemes: The Urban Employee Basic Medical Insurance (UEBMI) and the Urban and Rural Resident Basic Medical Insurance (URRBMI). We analyzed three key indicators: deductible threshold, reimbursement ratio, and reimbursement cap, using descriptive statistics to assess disparities and correlate them with local GDP per capita.

**Results:**

Significant differences were observed in reimbursement caps and ratios between the two medical insurance schemes (*P* < 0.005 and *P* < 0.001, respectively). Only the reimbursement cap was positively correlated with GDP per capita under both schemes (rs = 0.27, *P* = 0.01 for UEBMI; rs = 0.36, *P* = 0.001 for URRBMI). Longitudinally, sample cities showed unchanged or lower deductible thresholds, while reimbursement ratios and caps either remained unchanged or increased.

**Conclusions:**

National guidelines for outpatient cancer reimbursement are needed in China to facilitate local policy refinement and enhance outpatient reimbursement level, thereby promoting healthcare accessibility and equity.

**Supplementary Information:**

The online version contains supplementary material available at 10.1186/s12913-026-14418-0.

## Background

Cancer is a leading cause of mortality and morbidity worldwide, accounting for a substantial proportion of global disease burden. According to the World Health Organization (WHO), cancer is the first or second leading cause of death in individuals aged < 70 years in most countries [1]. In 2020, nearly 10 million people died from cancer-related illnesses globally [2]. By 2040, the annual number of new cancer cases worldwide will reach 29.5 million, with cancer-related deaths rising to 16.5 million [3]. Notably, cancer has been the primary cause of death among the Chinese population since 2010 [4]. In 2020, China accounted for 24.0% of new global cancer cases and 30.2% of cancer-related deaths, far surpassing other countries [5].

The cost of cancer treatment has risen exponentially, reaching USD 193 billion globally in 2022 and projected to exceed USD 370 billion by 2027 [6]. In low- and middle-income countries (LMICs), the high cost of anticancer medications obstructs access to effective and timely cancer treatment for most patients [7]. Even in high-income countries (HICs), the substantial cost of cancer treatment also poses a challenge. For instance, the average cost of cancer treatment in the United Kingdom increased tenfold over the two decades following 1995 [8]. In China, annual cancer treatment expenses exceed RMB 220 billion, with medications accounting for approximately 50% of total costs [9, 10]. These high costs impose significant financial strain on both patients and healthcare systems.

China achieved universal health insurance coverage (UHIC) in 2011 [9], and the basic medical insurance (BMI) program has played a significant role in reducing economic burdens and improving health outcomes [10]. Initially, this program primarily focused on inpatient care, excluding outpatient service. Consequently, for chronic patients, particularly those with conditions like cancer, a substantial portion of healthcare expenditures comes from outpatient medications, which were not covered by the BMI program. Over the past decade, China has prioritized enhancing outpatient reimbursement policies for chronic diseases, including cancer, as part of the “14th Five-Year Plan for Medical Insurance System with Universal Coverage [11]”.

The National Healthcare Security Administration mandates that each prefecture-level city integrate outpatient expenses for chronic and specific diseases into their reimbursement frameworks. This integration is contingent upon the financial capacity of their respective BMI funds and provincial-specific conditions. Given the substantial disease burden associated with cancer among chronic diseases, several provinces have formulated targeted policies for outpatient radiotherapy and chemotherapy services, such as Hunan Province’s “Regulations on Medical Insurance Reimbursement for Outpatient Radiotherapy and Chemotherapy for Cancer [12]”, aimed at optimizing medical resource utilization and curbing overall expenditure. However, this policy-making process, while allowing for local fiscal adaptability, may pose a risk to horizontal equity, where patients with identical clinical needs may face different financial burdens that are not systematically aligned with local economic capacity, depending on their geographical location or insurance program.

To analyze these disparities, we employ the three fundamental metrics officially mandated by national regulations [13] to define the BMI benefit package—the deductible threshold, reimbursement ratio, and reimbursement cap—as our primary quantitative tools. Based on this policy structure, we hypothesize that higher deductible thresholds have a negative impact on equity by raising the financial barrier to initial access; conversely, higher reimbursement ratios and higher caps are hypothesized to have a positive impact by reducing the patient’s co-payment burden.

Consequently, this study investigates the current status, disparities, and evolution of outpatient reimbursement policies for cancer treatment across different regions and medical insurance schemes in China. A cross-sectional analysis was conducted in 2022, supplemented by a longitudinal policy analysis.

## Methods

### Study Site

In the cross-sectional study of outpatient reimbursement policies for cancers in China, we included all four municipalities directly under the central government: Beijing, Shanghai, Tianjin, and Chongqing. For the remaining 22 provinces (excluding Taiwan, Hongkong and Macao) and five autonomous regions, we identified the provincial capitals and selected two additional cities from each with the highest and lowest GDP per capita in 2022 (excluding the provincial capitals themselves). We employed this stratification to implement a ‘maximum variation sampling’ strategy. We used GDP per capita as the principal metric because China’s Basic Medical Insurance (BMI) funds are mainly managed at the prefecture level [14, 15], and their fundraising capacity is heavily dependent on the local economic level and fiscal revenue. This allowed us to observe the maximum potential inequality within each province. Our sample ultimately comprised 85 prefecture-level cities (see Table S1).

Furthermore, we selected provincial capitals and municipalities directly under the central government with the highest and lowest GDP per capita in 2022 within the eastern, central, and western regions of China (a total of six prefecture-level cities). This selection criterion was designed to capture the temporal evolution of policy disparities across China’s three major economic belts. By tracking the cities with the most and least robust economic foundations within each region, we aimed to trace the evolution of their outpatient reimbursement policies for cancers in 2015 and 2019 (see Table S2).

### Data source

We systematically searched the official websites of the medical insurance bureau, finance bureaus, and people’s governments of the selected prefecture-level cities, using keywords such as “chronic disease” and “chronic disease clinic”. We extracted the GDP per capita for each prefecture-level city in 2022 from the 2022 China City Statistical Yearbook, published by the Department of Urban Surveys of the National Bureau of Statistics. To ensure data accuracy, consistency, and inter-rater reliability, two independent researchers extracted key policy information—including deductible thresholds, reimbursement ratios, and reimbursement caps — from official government documents and public notice websites. In cases where policy descriptions were ambiguous or inconsistent, the researchers first attempted to reach a consensus through discussion; if consensus could not be achieved, the discrepancies were resolved by a third senior research (CaiJun Yang).

### Data analysis

Firstly, we summarized the deductible thresholds, reimbursement ratios, and reimbursement caps for outpatient reimbursements for chronic diseases in each city. Additionally, we examined the reimbursement processes and routes for outpatient reimbursements for cancers in these cities.

Secondly, we conducted a quantitative analysis of medical insurance reimbursement in China in 2022. We calculated the deductible thresholds, reimbursement ratios (using the median value when the ratio was an interval) and reimbursement cap for outpatient services under the two schemes**—** the Urban Employee Basic Medical Insurance (UEBMI) and the Urban and Rural Resident Basic Medical Insurance (URRBMI)**—** in each prefecture-level city. The UEBMI is designed for urban employed individuals, whereas the URRBMI is for urban and rural residents without formal employment (including children, the elderly, and other unemployed individuals). A paired-samples t-test was employed to compare the city-level differences between the UEBMI and URRBMI for each policy indicator, with UEBMI and URRBMI indicators paired within the same city. Furthermore, we explored the correlation between the reimbursement capability of medical insurance and the economic level among different prefecture-level cities. We also quantitatively compared the differences between outpatient and inpatient cancer reimbursement levels in various prefecture-level cities.

Lastly, a longitudinal analysis of medical insurance reimbursement in China was conducted by comparing the deductible threshold, reimbursement ratio, and reimbursement cap for outpatient cancer reimbursement over time (2015, 2019, and 2022) in the six sample cities.

## Results

### Outpatient reimbursement policies for cancer patients in 2022

#### Outpatient reimbursement policies for chronic diseases

A comprehensive summary of the deductible thresholds, reimbursement ratios, and reimbursement caps for outpatient reimbursements for chronic diseases across various prefecture-level cities in China is presented in Table S3. Nearly half of the cities (45.24%) did not establish a specific deductible threshold. Thirty-one cities (36.90%) uniformly set the threshold for all chronic diseases, while the remaining 15 cities (17.86%) established varied thresholds based on specific diseases.

When considering the reimbursement ratio, more than half of the cities (61.90%) adopted a uniform ratio for all chronic diseases. Twenty cities (23.81%) determined different reimbursement ratios based on the type of disease, while the remaining 12 cities (14.29%) stipulated that the reimbursement ratios should be set with reference to the reimbursement ratios for hospitalization.

In terms of reimbursement cap, 56 cities (66.67%) set different caps for different chronic diseases. Alternatively, 28 cities (33.33%) incorporated outpatient reimbursement caps for chronic diseases into their hospitalization reimbursement systems, thereby sharing the same reimbursement cap for hospitalization.

#### Reimbursement routes for outpatient cancer treatment

**Fig. 1:**
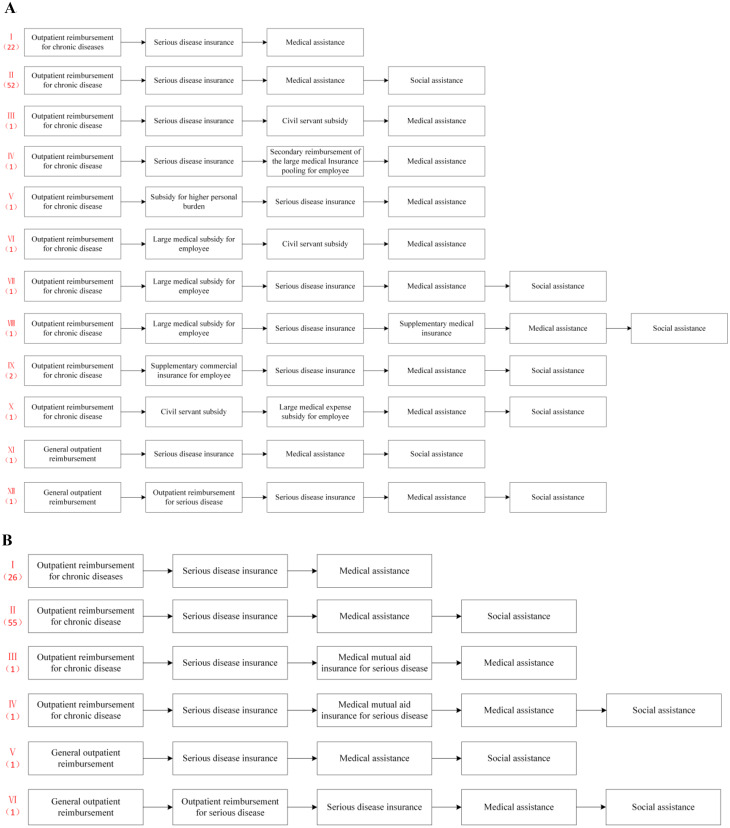
(**A**) Outpatient reimbursement routes for cancers under the UEBMI in sample cities. (**B**) Outpatient reimbursement routes for cancers under the URRBMI in sample cities

Figure 1 (including parts A and B) illustrates the variations in the full reimbursement routes for the outpatient treatment of cancers among patients enrolled in different medical insurance schemes.

Figure 1A summarizes a total of 12 outpatient reimbursement routes for cancers under the UEBMI in 85 sample cities, denoted by Route I-XII. Route I is the shortest reimbursement pathway, including three common processes. A total of 22 cities (25.88%) adopted this route. Route II, which adds “social assistance” to Route I, is utilized by 52 cities (61.18%), accounting for the highest proportion. Social assistance refers to the financial support or subsidies provided by governments or social welfare programs to individuals or families to help them cover the costs of healthcare services, such as the Anhui Cancer Foundation, Guizhou Red Cross Tumor Prevention and Control Foundation, and the Jilin Red Cross Tumor Assistance Project. Route VIII adopted by the Xinjiang-HE contains a total of six segments, which is the pathway that contains the largest number of items. Route XI is used in the Fujian-HE. As it lacks a specific outpatient policy for chronic and special diseases but offers an all-disease outpatient security system, outpatient coverage is available for all types of outpatient visits, regardless of the disease in the Fujian-HE. Shanghai uses Route XII, where patients with cancers first reimburse at the general outpatient clinic and then seek reimbursement for the remaining out-of-pocket expenses through the outpatient clinic for serious disease.

Figure 1B illustrates the outpatient reimbursement routes for cancers under the URRBMI in the sample cities. Compared with the UEBMI, the URRBMI has fewer reimbursement routes (only six). Among these, Routes I and II accounted for the highest proportions (30.59% and 64.71%, respectively). Similar to the pathway in the UEBMI, the Fujian-HE, which has implemented outpatient security for all disease types, is the only city to adopt Route V. Similarly, Shanghai is the sole city to adopt Route VI, which involves general outpatient reimbursement followed by reimbursement of out-of-pocket expenses through the outpatient clinic for serious disease.

Figure 1. Outpatient reimbursement pathways for cancers under different medical insurance schemes in various prefecture-level cities.

#### Quantitative analysis of outpatient reimbursement for cancer patients

##### Descriptive analysis of policy elements

Table 1 presents the deductible thresholds, reimbursement ratios, and reimbursement caps for outpatient cancer treatment under the UEBMI and URRBMI schemes across various cities.

Regarding deductible thresholds, over half of the prefecture-level cities under both schemes abolished the outpatient deductible thresholds, with proportions of 55.29% for UEBMI and 56.47% for URRBMI. Specifically, 22 cities under UEBMI and 16 under URRBMI have deductible thresholds of 500 yuan or more, while 5 cities under UEBMI and 6 cities under URRBMI have thresholds exceeding 1,000 yuan. There was no significant difference in the deductible thresholds for cancer treatment between the two schemes (*P* = 0.29).

In terms of reimbursement ratios, all cities under the UEBMI have ratios above 60%, whereas 29 cities (34.12% [95% CI: 24.1%–44.2%]) under the URRBMI have ratios of 60% or below. Notably, 62 cities (72.94% [95% CI: 63.5%–82.3%]) under UEBMI offer reimbursement ratios exceeding 81%, compared to only 6 cities (7.06% [95% CI: 1.6%–12.5%]) under URRBMI. No prefecture-level city under the URRBMI provided a reimbursement ratio above 90%. Significantly higher reimbursement ratios for outpatient cancer treatment were observed under UEBMI compared to URRBMI (*P* < 0.001).

Regarding reimbursement caps, one city under the UEBMI and two under the URRBMI have caps of 10,000 RMB or less. Conversely, 14 cities under UEBMI and 6 under URRBMI have caps exceeding 300,000 yuan. The outpatient reimbursement cap for cancer treatment under UEBMI is also significantly higher than that under URRBMI (*P* < 0.005).

**Table 1:** Deductible thresholds, reimbursement ratios, and reimbursement caps for outpatient reimbursement of cancers under different medical insurance schemes

Policy elements	Number of cities(%)		*p*-value
	UEBMI	URRBMI	
Deductible Threshold			
0	47(55.29%)	48(56.47%)	0.29
1-100	3(3.53%)	3(3.53%)	
101–500	13(15.29%)	18(21.18%)	
501–1000	17(20.00%)	10(11.76%)	
> 1000	5(5.86%)	6(7.06%)	
Reimbursement Ratio			
50%-60%	0(0%)	29(34.12%)	< 0.001
61%-70%	3(3.53%)	30(35.29%)	
71%-80%	20(23.53%)	20(23.53%)	
81%-90%	53(62.35%)	6(7.06%)	
> 90%	9(10.59%)	0(0%)	
Reimbursement Cap			
≤ 10,000	1(1.18%)	2(2.35%)	< 0.005
10,001–100,000	34(40.00%)	28(32.94%)	
100,000–200,000	23(27.06%)	37(43.53%)	
200,001–300,000	13(15.29%)	12(14.12%)	
300,001–400,000	2(2.35%)	4(4.71%)	
400,001–500,000	5(5.88%)	1(1.18%)	
> 500,000	7(8.24%)	1(1.18%)	

##### Relationship between policy elements and GDP per capita

**Fig. 2:**
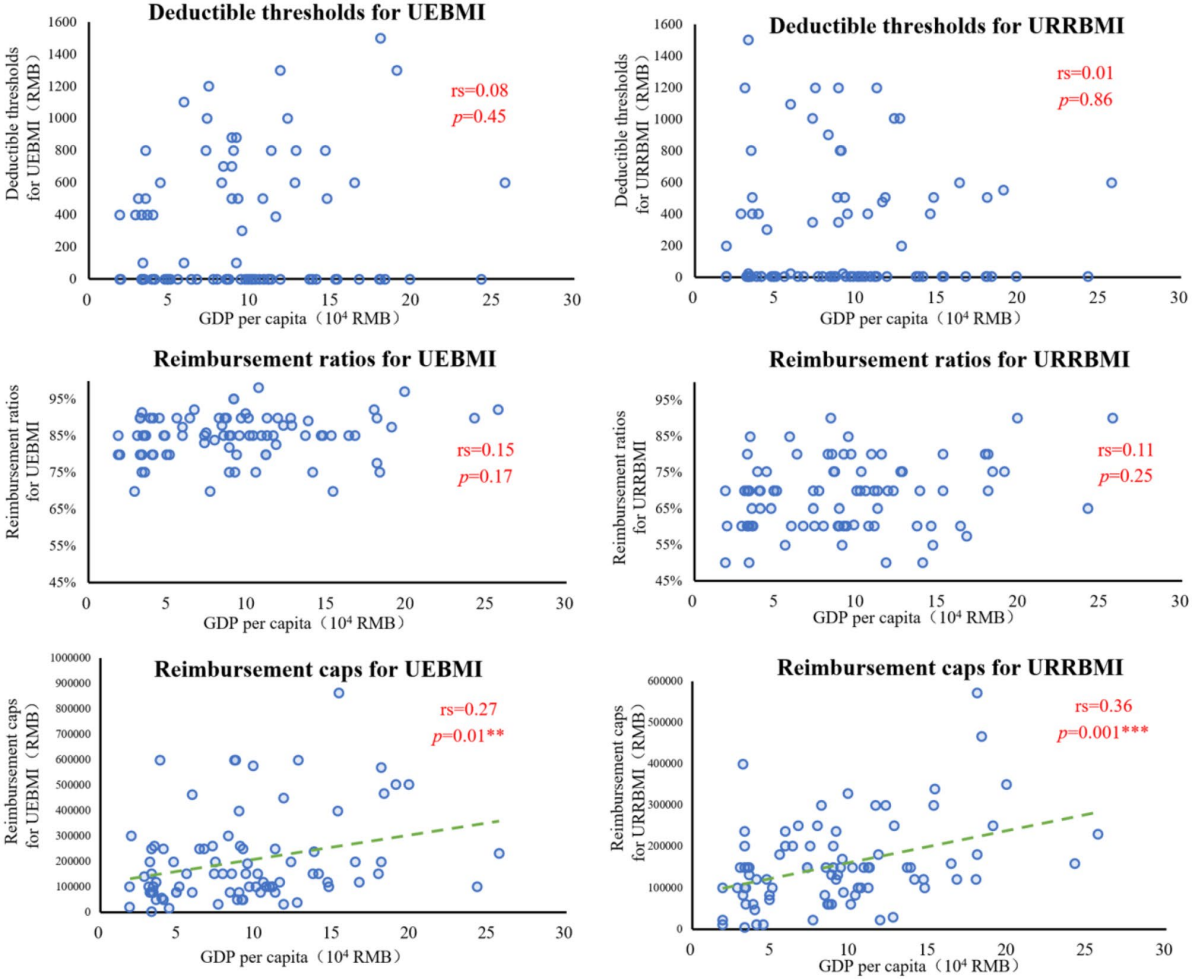
Correlation of deductible thresholds, reimbursement rates, reimbursement limits and GDP per capita for different medical insurance schemes

To investigate potential disparities in medical insurance reimbursement for outpatient cancer treatment across cities, the Spearman correlation coefficient was employed to assess the relationship between deductible thresholds, reimbursement ratios, reimbursement caps under the two medical insurance schemes, and local GDP per capita. The results (see Fig. 2) indicated no statistically significant correlation between GDP per capita and either the deductible thresholds for UEBMI (*P* = 0.45) or URRBMI (*P* = 0.86). Similarly, no significant correlation was observed between GDP per capita and the reimbursement ratios for UEBMI (*P* = 0.17) or URRBMI (*P* = 0.25). However, a statistically significant positive correlation was found between GDP per capita and reimbursement caps for both UEBMI (*r* = 0.27, *P* = 0.01) and URRBMI (*r* = 0.36, *P* = 0.001) in prefecture-level cities.

##### Optimal policy point for patients

**Fig. 3:**
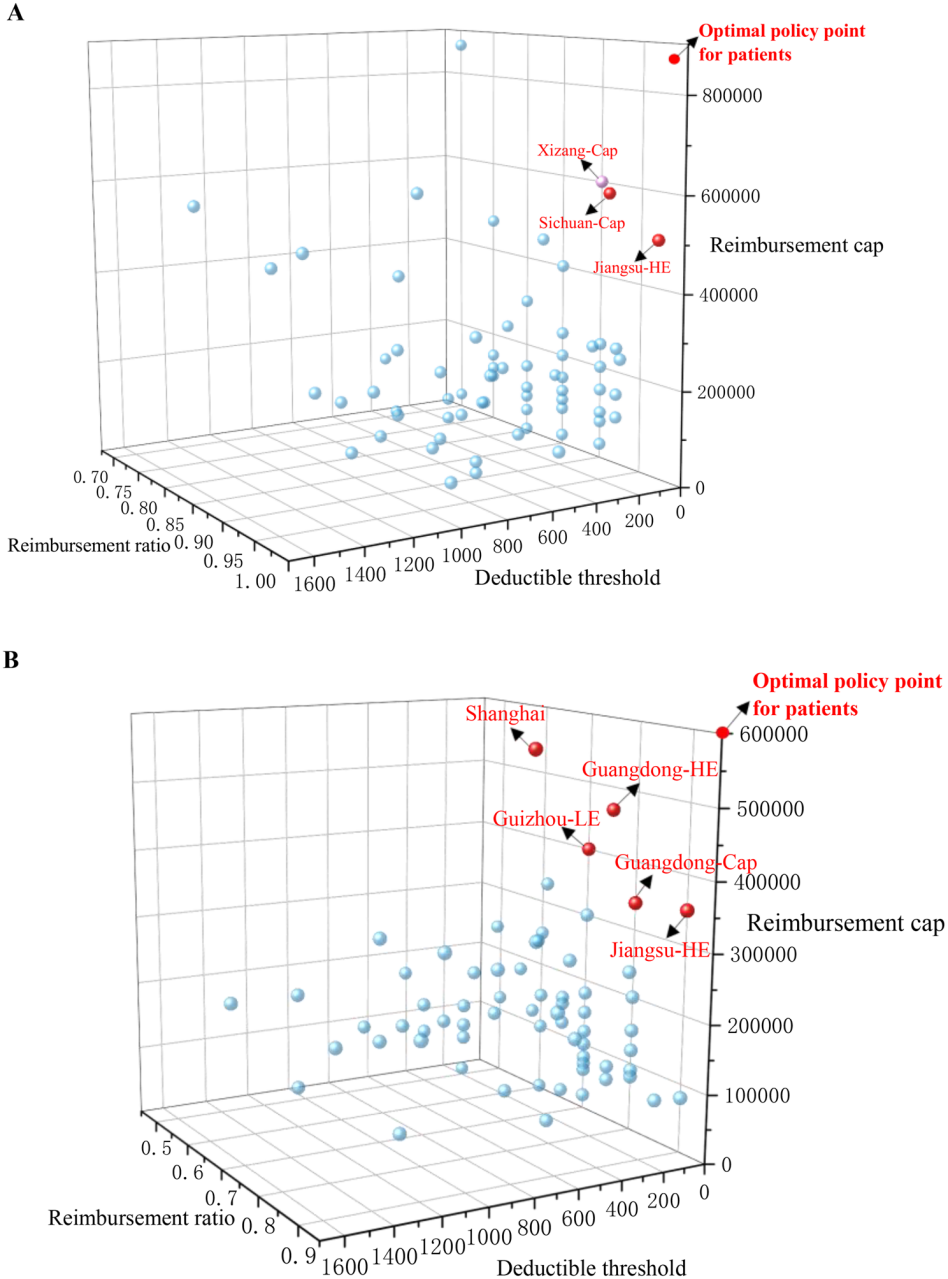
(**A**) Three-dimensional scatterplot of outpatient reimbursement for cancer under the UEBMI in sample cities. (**B**) Three-dimensional scatterplot of outpatient reimbursement for cancer under the URRBMI in sample cities

Three-dimensional scatterplots were constructed to evaluate outpatient reimbursement efforts for cancers under UEBMI and URRBMI across cities, as depicted in Fig. 3. In these scatterplots, proximity to the “optimal policy point for patients” (characterized by the lowest deductible threshold, highest reimbursement ratio, and highest reimbursement cap) indicates more effective policy implementation from the patient’s perspective, rather than that of the healthcare system. Figure 3A illustrates the outpatient reimbursement policies for cancers under the UEBMI in various cities, with the Xizang-Cap, the Sichuan-Cap, and the Jiangsu-HE being closest to the optimal point. Similarly, Fig. 3B shows the corresponding situation for the URRBMI, with Shanghai, the Guangdong-HE, the Guizhou-LE, the Guangdong-Cap, and the Jiangsu-HE nearest to the optimal point. Therefore, Wuxi is the only city with an optimal reimbursement policy for patients under both UEBMI and URRBMI schemes.

Figure 3 Scatterplots of outpatient reimbursement for cancers under different medical insurance schemes in various prefecture-level cities.

##### Comparison of outpatient versus inpatient reimbursement

Quantitative comparison of outpatient versus inpatient reimbursement for cancers in local tertiary hospitals across various cities are presented in Table S4. Outpatient reimbursement exhibited distinct characteristics across various cities compared with inpatient reimbursement under the UEBMI scheme. Specifically, in two cities (2.35%), namely the Anhui-HE and the Fujian-LE, the deductible thresholds for outpatient reimbursements were higher. Additionally, the reimbursement ratios for outpatient services were lower in 27 cities (31.76%). Furthermore, outpatient reimbursements in 13 cities (15.29%) demonstrated lower reimbursement caps. Similarly, under the URRBMI scheme, outpatient reimbursements also showed notable variations. In 4 cities (4.71%), the deductible thresholds for outpatient reimbursements were higher. The reimbursement ratios were lower in 14 cities (16.47%). Moreover, in 12 cities (14.12%), the reimbursement caps were lower.

### Outpatient reimbursement policies for cancer patients over time

Table 2 demonstrates the variations in outpatient reimbursement indicators for cancer treatment in selected cities across eastern, central, and western China in 2015, 2019, and 2022, respectively.

Under the UEBMI scheme, it is noteworthy that the Hebei-Cap abolished its outpatient deductible threshold of 600 RMB in 2015, whereas the thresholds remained unchanged in the other five cities. Regarding the reimbursement ratios, all five cities observed an increase, with the exception of Beijing. Notably, the Xinjiang-Cap experienced a significant 20% rise in its reimbursement ratio. In terms of reimbursement caps, five cities witnessed an increase, with the Hubei-Cap maintaining its cap of 240,000 RMB. It is particularly worth mentioning that Xining’s reimbursement cap increased nine-fold, from 10,000 RMB to 100,000 RMB.

Under the URRBMI scheme, half of the sampled cities, namely the Xinjiang-Cap, the Hebei-Cap, and the Qinghai-Cap, reduced their outpatient deductible thresholds. Across all six cities, the reimbursement ratios increased but remained lower compared to those under the UEBMI scheme. Regarding reimbursement caps, all cities except the Xinjiang-Cap (unchanged) observed an increase.

**Table 2:** Changes in outpatient reimbursement for cancers in sample cities

Sample Cities	UEBMI				URRBMI			
	2015	2019	2022	Changes	2015	2019	2022	Changes
Deductible Threshold								
Beijing	1300	1300	1300	Unchanged	1300	1300	1300	Unchanged
Hubei-Cap	0	0	0	Unchanged	0	0	0	Unchanged
Xinjiang-Cap	0	0	0	Unchanged	10	0	0	Decreased
Hebei-Cap	600	0	0	Decreased	900	0	0	Decreased
Heilongjiang-Cap	0	0	0	Unchanged	0	0	0	Unchanged
Qinghai-Cap	0	0	0	Unchanged	200	200	0	Decreased
Reimbursement Ratio								
Beijing	87.5%	87.5%	87.5%	Unchanged	70.0%	75.0%	75.0%	Increased
Hubei-Cap	87.0%	87.0%	89.0%	Increased	50.0%	50.0%	70.0%	Increased
Xinjiang-Cap	70.0%	90.0%	90.0%	Increased	60.0%	80.0%	80.0%	Increased
Hebei-Cap	87.5%	90.0%	90.0%	Increased	60.0%	80.0%	80.0%	Increased
Heilongjiang-Cap	80.0%	90.0%	90.0%	Increased	50.0%	52.5%	55.0%	Increased
Qinghai-Cap	65.0%	65.0%	75.0%	Increased	50.0%	50.0%	70.0%	Increased
Reimbursement Cap								
Beijing	300,000	300,000	500,000	Increased	170,000	200,000	250,000	Increased
Hubei-Cap	240,000	240,000	240,000	Unchanged	130,000	150,000	150,000	Increased
Xinjiang-Cap	80,000	80,000	100,000	Increased	90,000	90,000	90,000	Unchanged
Hebei-Cap	200,000	250,000	250,000	Increased	120,000	200,000	200,000	Increased
Heilongjiang-Cap	100,000	100,000	150,000	Increased	110,000	170,000	180,000	Increased
Qinghai-Cap	10,000	10,000	100,000	Increased	10,000	10,000	100,000	Increased

## Discussion

The current outpatient reimbursement policy for chronic diseases in China faces significant challenges due to the absence of a national-level design. Integration and harmonization of chronic disease outpatient reimbursement policies can help achieve reform and improvement of outpatient services for chronic diseases [16]. Notably, significant disparities existed in the scope and number of outpatient services for cancer treatment, both across cities and within the same city, between the two medical insurance schemes (UEBMI and URRBMI). These disparities hinder the portability and continuity of BMI, thereby impeding equitable access to healthcare and obstructing the development of BMI program. Considering the economic and epidemiological diversity among cities, it is impractical to establish a uniform national outpatient coverage for cancers. Therefore, a national-level framework and guiding principles for outpatient access to cancer treatment should first be established. Subsequently, provincial governments can tailor the specific disease scope and access criteria according to their economic conditions and fund-payment capacities. Since local governments bear the ultimate responsibility for insurance fund solvency under China’s decentralized fiscal system, a rigid ‘one-size-fits-all’ national mandate could jeopardize financial sustainability in less developed regions and trigger local resistance -undermining political feasibility. Therefore, the central government should establish clear ‘minimum benefit floors’ to guarantee basic equity across regions and set a uniform baseline for protection. Meanwhile, local authorities should retain the flexibility to adjust benefit standards above the floor based on their fiscal capacity, reconciling the trade-off between equity and sustainability while balancing central coordination with local autonomy.

China has implemented diversified reimbursement channels for the medical expenses of cancer patients to alleviate their financial burden, yet significant regional and insurance-scheme variations persist in reimbursement pathways. Unlike other policies centralized by the central government and decentralized for local implementation, the outpatient reimbursement system for cancer is primarily developed provincially. However, only a few provinces, such as Hunan, with its Interim Measures for Medical Insurance Payment for Outpatient Radiotherapy and Chemotherapy of Cancers [12], and Shanghai with its Notice on Matters Relating to the Improvement of Outpatient Medical Policies for Major Diseases of Malignant Tumors and Management of Medical Insurance in the City [17], have introduced relevant policies, resulting in a fragmented security system. The extensive variation in reimbursement pathways identified in this study reflects the fragmentation of the current healthcare insurance system. While diversified channels are intended to provide multi-layered protection, the absence of a unified national framework means that a patient’s administrative experience is largely determined by their place of residency.

Our analysis revealed that while reimbursement caps exhibited a weak positive correlation with local GDP per capita, no statistically significant associations were observed for deductible thresholds or reimbursement ratios. This pattern indicates that key reimbursement parameters are not consistently aligned with local economic capacity. Such misalignment warrants caution with respect to potential horizontal inequity, as patients residing in cities with comparable economic conditions may nonetheless experience markedly different financial burdens due to variations in reimbursement standards.

In our three-dimensional analysis, we utilized the ‘Optimal Policy Point’ (characterized by the lowest deductible threshold, highest reimbursement ratio, and highest reimbursement cap) as a benchmark. It is important to note that while this point represents the scenario for minimizing patients’ financial burden, it does not equate to the “global optimum” for the healthcare insurance system. From a health policy perspective, setting deductibles and co-payment ratios is a necessary measure to ensure the long-term sustainability of insurance funds. This study has not conducted an empirical analysis of the actual financial sustainability of each city’s policy. Therefore, the proximity of a city’s policy to this ideal point should be interpreted solely as a metric for measuring the strength of financial protection offered to cancer patients. Cities closest to this point (e.g., Wuxi) demonstrate a strong policy orientation toward prioritizing affordability within the limits of their fiscal capacity.

In light of these findings, we recommend a two-tiered improvement strategy. First, at the national level, clearer guidelines should be established to encourage local authorities to link reimbursement standards to indicators of local affordability, such as disposable income. Second, at the local level, governments should actively benchmark their benefit designs against cities of similar developmental status that demonstrate superior performance in our three-dimensional analysis. Together, these measures may help reduce arbitrary variation in benefit design and enhance the overall equity of the medical security system.

In some cities, outpatient reimbursement for cancers falls below that for inpatient care. Given that cancer treatment primarily relies on medication, outpatient treatment offers enhanced convenience, reduced economic burden, and lessened psychological stress to patients. If outpatient reimbursement for cancer is inferior to inpatient reimbursement, patients may delay treatment due to cost concerns or opt for inpatient care solely to obtain compensation [18], which is detrimental to early disease control and results in a dual waste of medical resources and insurance funds. For example, Miao et al. [19, 20] intervened and controlled outpatient costs for thousands of hypertensive patients in two counties in Hubei Province, China, and proposed that outpatient expenditures should be reimbursed at a higher ratio to alleviate reliance on inpatient hospital services by motivating outpatient services. Using administrative insurance claims data from UEBMI, Shen [21] et al. argued that increasing outpatient reimbursement limits for patients with hypertension and diabetes may be an important strategy for reducing adverse health events (e.g., hospitalizations) in China. The study by Du [22] et al. also suggested that decreasing the out-of-pocket expenses for outpatient care patients with diabetes have more rational health behaviors, better health status and more reasonable medical expenses while the expenditure of the basic medical insurance funds is stable in total. Therefore, it is recommended that local authorities, while ensuring the security of medical insurance funds, gradually bridge the reimbursement gap between outpatient and inpatient cancer treatment.

With the deepening reform of China’s BMI program, localities are gradually exploring and expanding the scope of outpatient chronic diseases in accordance with the funds’ ability to pay. Some specialist treatments that need to be carried out on an outpatient basis and are more cost-effective than inpatient hospitalization can be managed with reference to inpatient treatment [23]. Between 2015 and 2022, most sample cities in China observed increases in both the reimbursement ratios and caps for cancer outpatient treatment. However, a few cities remained unchanged. Consequently, on the premise of ensuring medical insurance fund security, cities should gradually enhance reimbursements for cancer treatment to better protect patients’ rights.

This study has some limitations. Firstly, three cities in each province of China (the capital city, the city with the highest and the lowest economic level) were selected as the sample in 2022, which is not yet comprehensively representative of outpatient cancer reimbursement in that province. Secondly, the study did not include patient-level data, such as utilization rates and out-of-pocket costs. This limits the ability to assess the real-world impact of the reimbursement policies on patients. It is important to note that this study is based solely on policy documents and official reimbursement standards. The ‘administrative burden’ and ‘financial protection’ discussed in this paper are theoretical inferences derived from policy design, rather than empirical observations of real-world patient experiences. Thirdly, due to the constraints of our sample size, this study primarily employed descriptive statistics and bivariate correlation analyses. While this approach effectively identifies the existence and magnitude of regional disparities—fulfilling the study’s monitoring objective—it does not allow for causal inference regarding the determinants of policy design. However, given the paucity of references on outpatient reimbursement policies for chronic diseases such as cancer in China, this study may be complementary to the field.

## Conclusion

In the face of varying outpatient cancer reimbursement in different cities and regions, China should establish national-level guidelines for outpatient cancer reimbursement, allowing provincial and municipal governments to improve specific policies according to local conditions, and to learn from the excellent reimbursement policies of other cities with similar levels of development, so as to help achieve fairness in healthcare protection.

The study’s insights into the evolution of reimbursement policies over time, with unchanged or lower deductible thresholds and increased reimbursement ratios and caps, provide a valuable reference for other developing nations striving to achieve universal health coverage under fiscal constraints. By adopting similar strategies, policy makers can enhance the financial protection for cancer patients, improve access to cancer treatment, and contribute to better health outcomes and a more sustainable healthcare system globally.

## Supplementary Information

Below is the link to the electronic supplementary material.

Supplementary Material 1

## Data Availability

The datasets used and/or analyzed during the current study are available from the corresponding author on reasonable request.

## Abbreviations

BMI: Basic Medical Insurance
UEBMI: Urban Employee Basic Medical Insurance
URRBMI: Urban and Rural Resident Basic Medical Insurance
GDP: Gross Domestic Product
WHO: World Health Organization
LMICs: Low- and middle-income countries
HICs: High-income countries
UHIC: Universal health insurance coverage
