# Supplementary material for: Outpatient reimbursement policies for cancer treatment in China: a comparative and longitudinal analysis
Source: BMC Health Serv Res. 2026 Mar 23;26:614. doi: 10.1186/s12913-026-14418-0 (PMC13130447; doi:10.1186/s12913-026-14418-0)
Supplement: Supplementary file 1 — Supplementary Material 1 [file 12913_2026_14418_MOESM1_ESM.docx]

Appendix

Table S1 Information on the sample cities included in the cross-sectional study of outpatient reimbursement policies for cancers

| Sample cities | GDP per capita in 2022/ 10^4^ RMB | Reason for inclusion and  abbreviation( Province-*) |
| --- | --- | --- |
| Beijing | 19.03 | Municipality directly under the central government |
| Chongqing | 9.07 | Municipality directly under the central government |
| Shanghai | 18.05 | Municipality directly under the central government |
| Tianjin | 11.79 | Municipality directly under the central government |
| Hefei | 12.69 | Anhui-Cap |
| Wuhu | 12.27 | Anhui-HE |
| Fuyang | 3.96 | Anhui-LE |
| Fuzhou | 14.59 | Fujian-Cap |
| Xiamen | 14.69 | Fujian-HE |
| Nanping | 8.34 | Fujian-LE |
| Lanzhou | 7.63 | Gansu-Cap |
| Jinchang | 11.86 | Gansu-HE |
| Linxia | 1.90 | Gansu-LE |
| Guangzhou | 15.33 | Guangdong-Cap |
| Shenzhen | 18.32 | Guangdong-HE |
| Meizhou | 3.43 | Guangdong-LE |
| Nanning | 5.91 | Guangxi-Cap |
| fangchenggang | 9.15 | Guangxi-HE |
| Hechi | 3.32 | Guangxi-LE |
| Guiyang | 7.91 | Guizhou-Cap |
| Zunyi | 6.67 | Guizhou-HE |
| Bijie | 3.23 | Guizhou-LE |
| Haikou | 7.26 | Hainan-Cap |
| Danzhou | 8.96 | Hainan-HE |
| Wuzhishan City | 3.49 | Hainan-LE |
| Shijiazhuang | 6.34 | Hebei-Cap |
| Tangshan | 11.56 | Hebei-HE |
| Xingtai | 3.58 | Hebei-LE |
| Zhengzhou | 10.15 | Henan-Cap |
| Jiyuan | 11.09 | Henan-HE |
| Zhoukou | 4.09 | Henan-LE |
| Harbin | 5.55 | Heilongjiang-Cap |
| Daqing | 10.87 | Heilongjiang-HE |
| Qiqihar | 3.31 | Heilongjiang-LE |
| Wuhan | 13.82 | Hubei-Cap |
| Yichang | 14.07 | Hubei-HE |
| Enshi | 4.06 | Hubei-LE |
| Changsha | 13.64 | Hunan-Cap |
| Yueyang | 9.34 | Hunan-HE |
| Xiangxi | 3.29 | Hunan-LE |
| Changchun | 7.41 | Jilin-Cap |
| Baishan | 5.88 | Jilin-HE |
| Siping | 3.28 | Jilin-LE |
| Nanjing | 17.94 | Jiangsu-Cap |
| Wuxi | 19.85 | Jiangsu-HE |
| Suqian | 8.22 | Jiangsu-LE |
| Nanchang | 11.1 | Jiangxi-Cap |
| Yingtan | 10.71 | Jiangxi-HE |
| Ganzhou | 5.04 | Jiangxi-LE |
| Shenyang | 8.44 | Liaoning-Cap |
| Dalian | 11.26 | Liaoning-HE |
| Tieling | 3.24 | Liaoning-LE |
| Hohhot | 9.44 | Inner Mongolia-Cap |
| Ordos | 25.69 | Inner Mongolia-HE |
| Xing'an League | 4.85 | Inner Mongolia-LE |
| Yinchuan | 8.80 | Ningxia-Cap |
| Shizuishan City | 9.21 | Ningxia-HE |
| Guyuan | 3.56 | Ningxia-LE |
| Xining | 3.35 | Qinghai-Cap |
| Haixi Prefecture | 3.37 | Qinghai-HE |
| Guoluo Prefecture | 1.92 | Qinghai-LE |
| Xi'an | 8.88 | Shaanxi-Cap |
| Yulin | 18.08 | Shaanxi-HE |
| Shangluo | 4.46 | Shaanxi-LE |
| Jinan | 12.77 | Shandong-Cap |
| Dongying | 16.39 | Shandong-HE |
| Liaocheng | 4.71 | Shandong-LE |
| Taiyuan | 10.29 | Shanxi-Cap |
| Jincheng | 10.53 | Shanxi-HE |
| Yuncheng | 4.87 | Shanxi-LE |
| Chengdu | 9.81 | Sichuan-Cap |
| Panzhihua | 10.03 | Sichuan-HE |
| Bazhong | 2.86 | Sichuan-LE |
| Lhasa | 8.59 | Xizang-Cap |
| Nyingchi | 8.68 | Xizang-HE |
| Nagqu City | 3.86 | Xizang-LE |
| Urumqi | 9.61 | Xinjiang-Cap |
| Karamay | 24.24 | Xinjiang-HE |
| Hotan | 1.96 | Xinjiang-LE |
| Kunming | 8.87 | Yunnan-Cap |
| Yuxi | 11.25 | Yunnan-HE |
| Zhaotong | 3.07 | Yunnan-LE |
| Hangzhou | 15.26 | Zhejiang-Cap |
| Zhoushan | 16.71 | Zhejiang-HE |
| Lishui | 7.28 | Zhejiang-LE |

Abbreviation：Cap, capital city (provincial capital cities); HE, high economic level city (cities with the highest GDP per capita in the province, excluding the provincial capital city); LE, low economic level city (cities with the lowest GDP per capita in the province, excluding the provincial capital city).

Table S2 Information on the sample cities included in a longitudinal study of the evolution of outpatient reimbursement policies for cancers

| Abbreviation | City | Reason for inclusion |
| --- | --- | --- |
| Beijing | Beijing | Municipality directly under the eastern government in the eastern China with the highest GDP per capita in 2022 |
| Hubei-Cap | Shijiazhuang | Provincial city in the eastern China with the lowest GDP per capita in 2022 |
| Xinjiang-Cap | Wuhan | Provincial city in the central China with the highest GDP per capita in 2022 |
| Hebei-Cap | Harbin | Provincial city in the central China with the lowest GDP per capita in 2022 |
| Heilongjiang-Cap | Urumqi | Provincial city in the western China with the highest GDP per capita in 2022 |
| Qinghai-Cap | Xining | Provincial city in the western China with the lowest GDP per capita in 2022 |

Table S3 Principles for setting the deductible thresholds, reimbursement ratios, and reimbursement caps for outpatient reimbursement for chronic disease across various prefecture-level cities

| Policy elements | Setting principles | Number of cities(%) |
| --- | --- | --- |
| Deductible threshold |  |  |
|  | No deductible threshold | 38（45.24%） |
|  | Setting uniformly | 31（36.90%） |
|  | Setting by disease type | 15（17.86%） |
| Reimbursement ratio |  |  |
|  | Setting uniformly | 52（61.90%） |
|  | Setting by disease type | 20（23.81%） |
|  | Reference to hospitalization | 12（14.29%） |
|  |  |  |
| Reimbursement cap |  |  |
|  | Setting by disease type | 56（66.67%） |
|  | Reference to and share it with hospitalization | 28（33.33%） |

Table S4 Comparison of outpatient reimbursement and inpatient reimbursement for cancers in various prefecture-level cities

| Policy elements | UEBMI | URRBMI |
| --- | --- | --- |
| Deductible threshold |  |  |
| Outpatient ＜ inpatient | 64（75.29%） | 69（81.18%） |
| Outpatient ＝ inpatient | 19（22.35%） | 12（14.12%） |
| Outpatient ＞ inpatient | 2（2.35%） | 4（4.71%） |
| Reimbursement ratio |  |  |
| Outpatient ＜ inpatient | 27（31.76%） | 14（16.47%） |
| Outpatient ＝ inpatient | 41（48.24%） | 36（42.35%） |
| Outpatient ＞ inpatient | 17（20.00%） | 35（41.18%） |
| Reimbursement cap |  |  |
| Outpatient ＜ inpatient | 13（15.29%） | 12（14.12%） |
| Outpatient ＝ inpatient | 72（84.71%） | 73（85.88%） |
